# Histomorphometrical Study on Regional Variation in Distribution of Sweat Glands in Buffalo Skin

**DOI:** 10.1155/2018/5345390

**Published:** 2018-08-16

**Authors:** Debajit Debbarma, Varinder Uppal, Neelam Bansal, Anuradha Gupta

**Affiliations:** Department of Veterinary Anatomy, College of Veterinary Science, Guru Angad Dev Veterinary and Animal Sciences University, Ludhiana 141004, Punjab, India

## Abstract

The study was conducted on skin of 24 buffaloes collected from slaughter house. The skin tissues were collected from dorsal, lateral, and ventral parts of head, neck, thorax, abdomen, and tail regions and fixed in 10% neutral buffered formalin. The tissues were processed for paraffin blocks preparation by acetone benzene schedule. The paraffin sections of 5-6 *μ*m were cut with rotary microtome and stained with hematoxylin and eosin. The sweat glands in buffaloes were of saccular and simple coiled tubular type. Most of the sweat glands were associated with hair follicles and consisted of a coiled secretory portion (body) and a straight duct. The secretory portion was made up of glandular tubules, myoepithelium, and basement membrane. The duct portion had a narrow lumen and was lined by simple cuboidal epithelium. The glandular epithelium was simple squamous, simple cuboidal, or low columnar type depending upon their stage of secretary activity. Two types of sweat glands were observed, i.e., apocrine and merocrine. Large number of blood vessels and nerve fibers were observed in the vicinity of the sweat glands. In head, neck, and tail regions the maximum number of sweat glands/mm^2^ was observed in dorsal side which did not vary significantly (p<0.05) from lateral and ventral side. In abdomen region the number of sweat glands/mm^2^ was maximum on lateral region which varied significantly from ventral region (p<0.05). Overall, the maximum number of sweat glands/mm^2^ was in head region followed by abdomen, thorax, neck, and tail but without any significant (p<0.05) difference. Maximum sweat gland diameter was found in abdomen region followed by thorax, head, neck, and tail region.

## 1. Introduction

The skin is one of the largest and the most important systems of the body and acts as a barrier between the external and internal environment [1]. It is a complex structure, being composed of different tissues. It is responsible for protection, thermoregulation, external sensory awareness, immunological defense, wound healing, perception, and excretion and is also an effective barrier which prevents desiccation of electrolytes and macromolecules from the body [2]. The skin is the multilayered organ comprising epidermis, dermis, and hypodermis and its layers get modified depending upon the species, habitat, and body region of the animal [3]. The hypodermis is crucial in controlling the temperature and nutrient storage, as well as secreting important hormones such as leptin [4].

Buffaloes are well adapted to diverse climatic conditions. Anatomically, buffalo skin is covered with a thick epidermis containing many melanin particles that give the skin surface its characteristic black colour. The melanin pigments trap ultraviolet rays and prevent them from penetrating through the dermis of the skin of lower tissue. Buffaloes exhibit great distress when exposed to direct solar radiation or when working in the sun during a hot weather. This is due to the fact that their bodies absorb a great deal of solar radiation because of dark skin and sparse coat of hair and in addition to that they possess a less efficient evaporative cooling system [5]. Also, histological knowledge of animal skin is very important to the tanner and leather chemist as it is important to know the changes that the structure undergoes when it is being converted into leather.

In literature histomorphochemical studies on skin are available in pig [6], sheep [7–11], goat [11, 12], camel [13], cattle, and buffalo [14–16] but scanty information is available regarding regional distribution of sweat glands in buffalo. So the present work was planned.

## 2. Materials and Methods

The study was conducted on skin of 24 buffaloes collected from slaughter house and postmortem hall of GADVASU, Ludhiana. The skin samples were collected from dorsal, lateral, and ventral regions of head, neck, thorax, abdomen, and tail. The tissues were fixed in 10% neutral buffered formalin. After the fixation, the tissues were processed for paraffin block preparation by acetone benzene schedule [17]. The blocks were prepared and sections of 5-6 *μ*m thickness were cut with rotary microtome. These paraffin sections were stained with hematoxylin and eosin. The micrometrical observations on number of sweat glands/mm^2^ and diameter of sebaceous glands were recorded in different regions of body on hematoxylin and eosin stained sections. The data obtained was statistically analyzed.

## 3. Results

The sweat glands in buffaloes were of saccular and simple coiled tubular type (Figures 5, 6, 7, 8, and 10). Most of the sweat glands were associated with hair follicles (Figures 5, 8, and 10). They were deeply situated into the reticular dermis. Buffalo sweat glands consisted of a coiled secretory portion (body) and a straight duct. The secretory portion was made up of glandular tubules, myoepithelium, and basement membrane. The myoepithelial cells were situated between the secretory cells and basement membrane (Figure 9). The duct portion had a narrow lumen and was lined by simple cuboidal epithelium. The upper ducts were lined by stratified squamous epithelium. The supranuclear cytoplasm of the cell was more eosinophilic. The glandular epithelium was simple squamous, simple cuboidal, or low columnar. In the present study two types of sweat glands were observed i.e., apocrine and merocrine. The free surface of the cells in apocrine sweat glands had cytoplasmic protrusion indicating secretory activity and the merocrine sweat glands were made of tubules of cuboidal or flattened cells. Large number of blood vessels and nerve fibers were observed in the vicinity of the sweat glands. Myoepithelial cells were located between the lining epithelium and the basement membrane. Elastic and collagen fibers were seen around the secretory portion of the glands. The sweat glands were mostly associated with primary hair follicles in upper rows whereas hair follicles in the deepest layer were devoid of sweat glands and were of secondary type.

**Figure 1:**
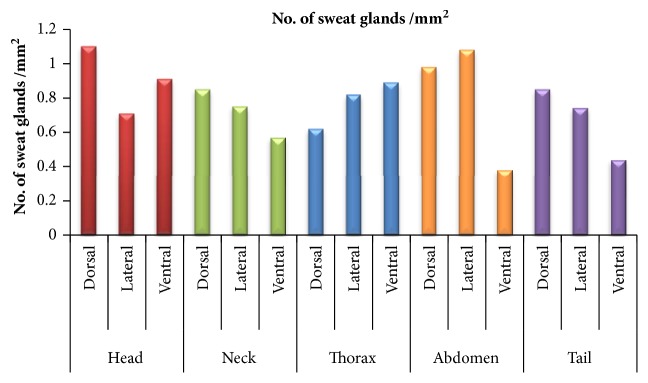
Graph showing distribution of no. of sweat glands/mm^2^ in different areas of body regions.

The distribution of sweat glands/mm^2^ in different body areas of different regions has been summarized in Table 1 and Figure 1. In head region the maximum number of sweat glands/mm^2^ was observed in head dorsal followed by head ventral and head lateral area but the difference was insignificant (p>0.05). In neck region, neck dorsal area had max. no. of sweat glands followed by neck lateral and neck ventral without any significant difference. In thorax region maximum number of sweat glands was observed in ventral area followed by lateral and dorsal areas without any significant difference (p>0.05). In abdomen maximum no. of sweat gland/mm^2^ was observed in lateral side followed by dorsal and ventral areas. The number of glands in abdomen ventral area was significantly (p<0.05) less than abdomen dorsal and abdomen lateral areas. In tail region glands were maximum in tail dorsal followed by lateral and ventral areas but without any significant difference (p>0.05).

**Figure 2:**
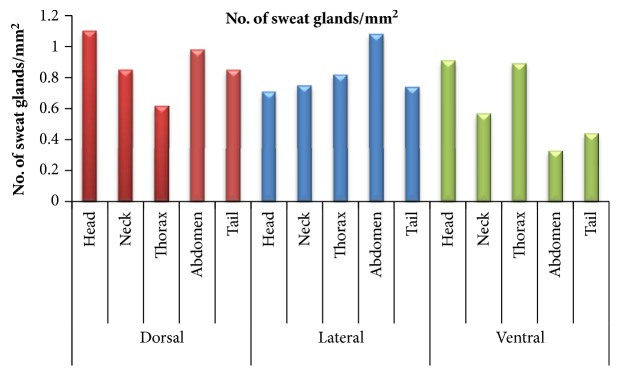
Graph showing distribution of no. of sweat glands/mm^2^ in different body regions.

When the distribution of no. of sweat glands/mm^2^ was observed among dorsal, lateral, and ventral areas of all regions, it was concluded that maximum number of sweat glands/mm^2^ was observed in head dorsal followed by abdomen dorsal, tail dorsal, neck dorsal, and thorax dorsal area without any significant difference (p>0.05). In lateral area among all the regions, maximum number of sweat gland was found in abdomen lateral region followed by thorax lateral, neck lateral, tail lateral, and head lateral region without any significant difference (p<0.05). In ventral side among all regions, maximum number of sweat glands/mm^2^ was observed in head ventral followed by thorax ventral, neck ventral, tail ventral, and abdomen ventral areas without any significant (p>0.05) difference. The distribution of number of sweat glands/mm2 in different body regions has been summarized in Table 2 and Figure 2. The maximum number of sweat glands/mm2 was in head region followed by abdomen, thorax, neck, and tail region without any significant difference (p>0.05).

**Figure 3:**
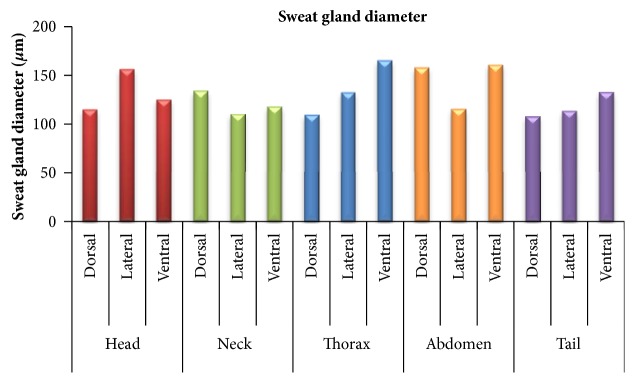
Graph showing diameter of sweat glands in different areas of body regions.

The diameter of sweat glands in different body areas of head, neck, thorax, abdomen, and tail has been summarized in Table 2 and Figure 3. In head region maximum diameter of sweat gland was observed in head lateral followed by head ventral areas with a significant difference (p<0.05) from head dorsal area. In neck region maximum diameter was in neck dorsal followed by neck ventral and neck lateral area but without any significant difference (p>0.05). In thorax maximum diameter was in ventral area followed by thorax lateral and thorax dorsal areas without any significant difference (p>0.05). In abdomen maximum diameter was in ventral area followed by abdomen dorsal and abdomen lateral. The diameter in abdomen ventral area was significantly higher (p<0.05) than diameter in abdomen lateral area. In tail region maximum diameter was in tail ventral followed by tail lateral area with a significant difference (p<0.05) from tail dorsal area.

A comparison of sweat gland diameter in dorsal, lateral, and ventral areas of all the body regions concluded that in dorsal area the maximum diameter was observed in abdomen dorsal followed by neck dorsal, head dorsal, thorax dorsal, and tail dorsal areas but the difference was significant (p<0.05) in the head, thorax, and tail. In lateral area the maximum diameter was observed in head lateral area followed by thorax lateral, abdomen lateral, tail lateral, and neck lateral areas. The difference was significant (p<0.05) in all the regions except thorax. In ventral area, maximum diameter was observed in thorax ventral followed by abdomen ventral, tail ventral, head ventral, and neck ventral with a significant difference (p<0.05) in head and neck region only.

**Figure 4:**
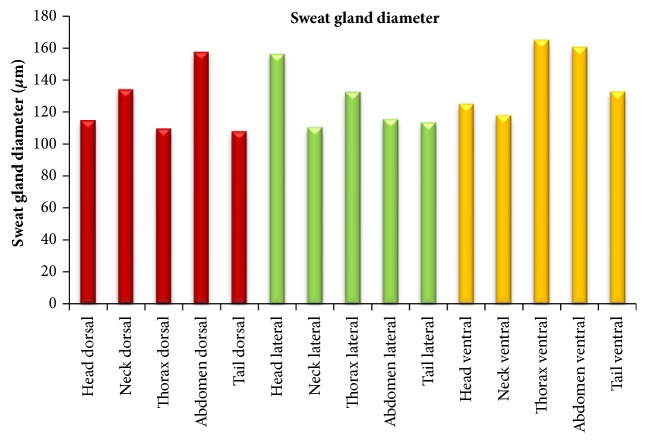
Graph showing a comparison of diameter of sweat glands in different areas of body regions.

**Figure 5:**
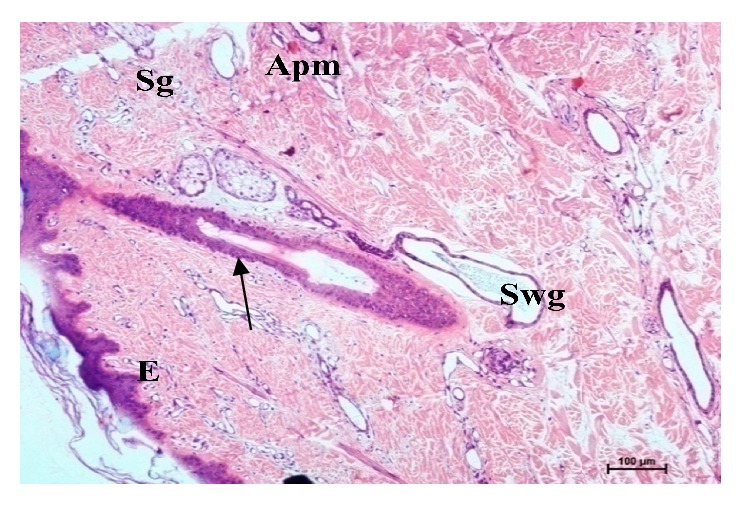
Photomicrographs from head ventral region skin showing epidermis (E), hair follicle (arrow), sweat gland (Swg), sebaceous gland (Sg), and arrector pili muscle (Apm). Hematoxylin and eosin X 100.

**Figure 6:**
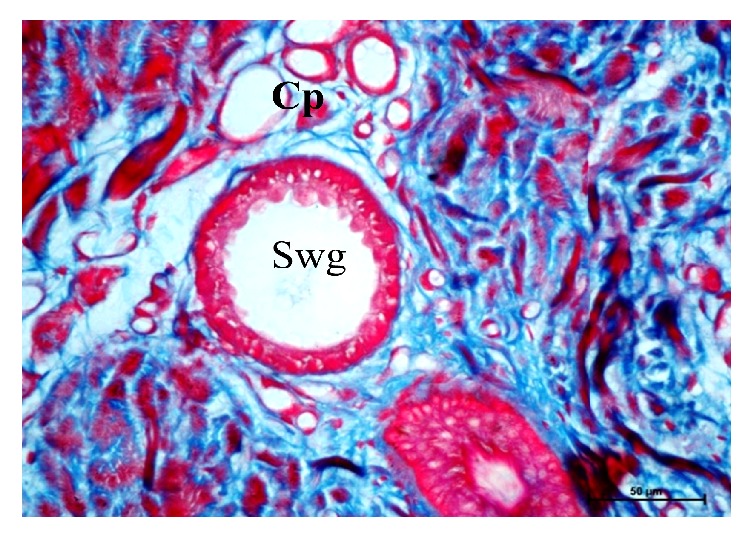
Photomicrographs from neck lateral region of skin showing sweat gland (Swg) and capillary Network (Cp). Masson, s trichrome X 400.

**Figure 7:**
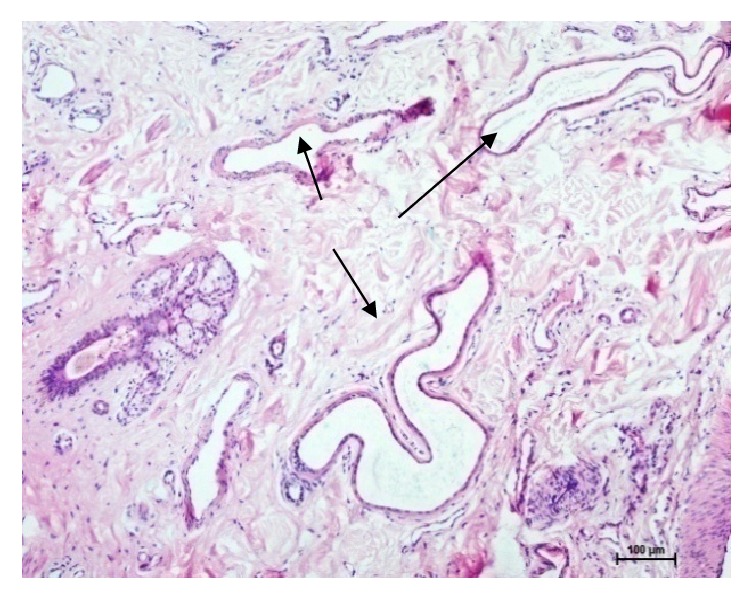
Photomicrographs from abdomen lateral region of skin showing sweat glands (arrow). Hematoxylin and eosin X 100.

**Figure 8:**
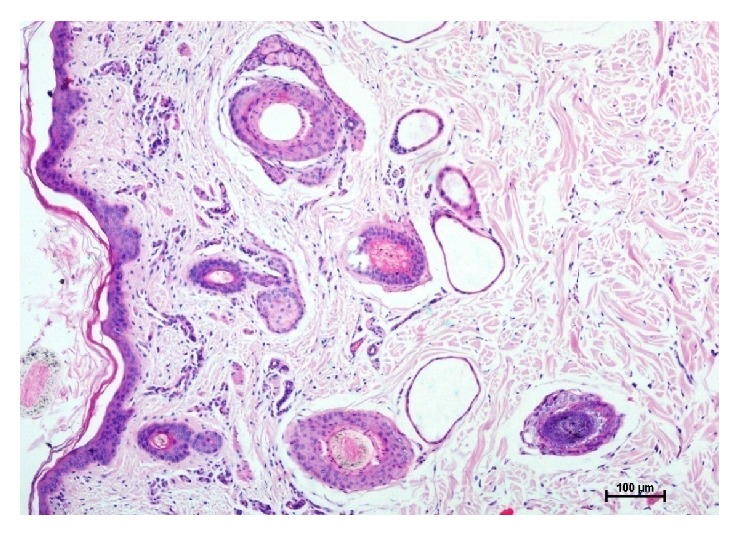
Photomicrographs from abdomen lateral region of skin showing epidermis (E), hair follicle (HF), sweat gland (Swg), and sebaceous gland (Sg). Hematoxylin and eosin X 100.

**Figure 9:**
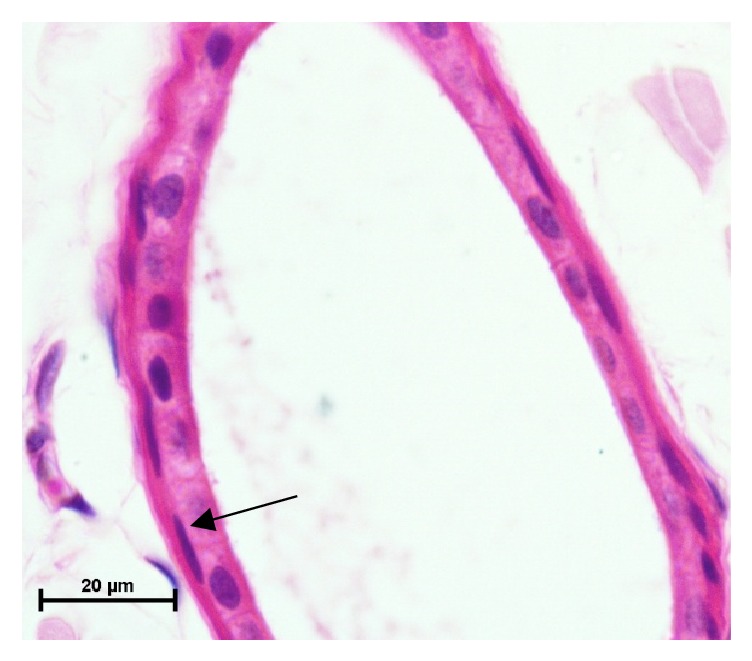
Photomicrograph from abdomen ventral region of skin showing myoepithelial cells (arrow) surrounding sweat gland. Hematoxylin and eosin X 1000.

**Figure 10:**
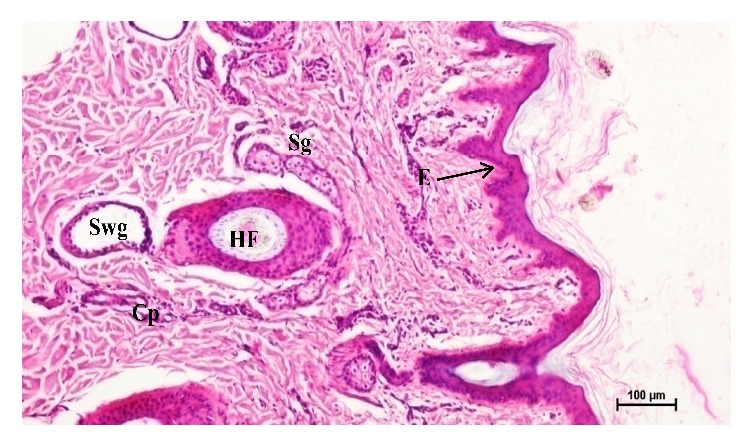
Photomicrograph from tail lateral region of skin showing Epidermis (E), hair follicle (HF), capillary network (Cp), sweat gland (Swg), and Sebaceous gland (Sg). Hematoxylin and Eosin X 100.

The sweat gland diameter in different body regions, i.e., head, neck, thorax, abdomen, and tail, has been summarized in Table 2 and Figure 4. The maximum sweat gland diameter was observed in abdomen region followed by thorax, head, neck, and tail but diameter in abdomen region was significantly higher (p<0.05) than in neck and tail regions.

## 4. Discussion

The sweat glands observed in present study were of saccular and simple coiled tubular type and most of them were associated with hair follicles as reported earlier by Taha and Abdalla [13], Goswami et al. [18] in camel, Schummer et al. [19] in domestic animals, Baba et al. [20] in sheep, and Razvi et al. [11] in goat. The glandular epithelium was the same as that observed by Hafez et al. [21] in buffalo and cattle, Goldsberry and Calhoun [22] and Govindaiah et al. [23] in cattle, Dowling and Nay [24] in camel, Lyne and Hollis [25], Bhayani et al. [7], and Mandage et al. [26] in sheep, Razvi et al. [11] in goat, Singh et al. [14] in Buffalo calves and Taha and Abdalla [13] in camel, and Sumena et al. [6] in pig. The glandular epithelium depends upon their stage of secretary activity [3]. Hafez et al. [21] in Buffalo and cattle, Goldsberry and Calhoun [22] and Govindaiah et al. [23] in cattle, Dowling and Nay [24] in camel, Lyne and Hollis [25] and Mandage et al. [26] in sheep, Singh et al. [14] in buffalo calves, and Taha and Abdalla [13] in camel have also reported two types of sweat glands similar to those observed in the present study. Buffalo skin has one-sixth of density of sweat glands that cattle skin has so buffaloes dissipate heat poorly by sweating as earlier reported by Marai and Haeeb [5] in Buffalo. Sweat glands were found in clusters and mainly associated with primary hair follicles in camel [18], in sheep [20], and in goat [11, 27]. In the present study, sweat glands were mainly associated with primary hair follicles as observed in camel [18], in sheep [20], and in goat [11, 27]. Saravanakumar and Thiagarajan [28] reported that the mean diameter was 156.7 *μ*m for Murrah, 155.3 *μ*m for Surti, and 157.7 *μ*m for nondescript type of buffaloes which was nearly in range with that of values of present study.

Govindaiah et al. [23] reported that number of sweat glands were more in neonatal cattle calves than young and adult animals. The depth of sweat glands increased with advancement of the age and highest density of sweat glands was observed in crossbred cattle below 12 months of age and lowest in adults of 96 months of age. These glands were maximum (10.40 ± 0.70) and minimum (2.10 ± 0.17) at dorsal and ventral regions of thorax, respectively, in neonatal age group. In young and adult age groups, the sweat glands were maximum at dorsal region of loin (5.00 ± 0.29 and 5.1 ± 0.27) and minimum in ventral region of thorax (1.60 ± 0.22 and 1.80 ± 0.20), respectively. Schummer et al. [19] observed that sweat glands were abundant in the skin of Large White Yorkshire pigs. At the neck dorsal and abdomen dorsal regions, the sweat glands were very large and lined by simple columnar epithelium whereas Sumena et al. [6] observed that the maximum number of sweat glands was observed in the snout region of Large White Yorkshire pigs. Razvi et al. [11] observed that density of sweat glands was maximum (10.40 ± 0.70) and minimum (2.10 ± 0.17) at dorsal and ventral regions of thorax, respectively. In young and adult age groups, the sweat glands were maximum at dorsal region of loin (5.00 ± 0.29 and 5.1 ± 0.27) and minimum at ventral region of thorax (1.60 ± 0.22 and 1.80 ± 0.20), respectively. Das et al. [29] reported in cattle that maximum number of sweat glands was in back region and minimum in abdomen. The maximum sweat gland diameter was observed in abdomen region followed by thorax, head, neck, and tail but difference was significantly higher (p<0.05) than in neck and tail regions only as reported by Patil et al. [30] in cattle and Das et al. [29] in cattle and Yak.

## Figures and Tables

**Table 1:** Number of sweat glands/mm^2^ and diameter of sweat glands in different body areas of different regions.

**Body region**	**Area**	**No. of Sweat glands /mm** ^**2**^	**Sweat gland diameter (** ***μ*** **m)**
Head	Dorsal	1.1 ± 0.1^a^	114.90 ± 5.97^b^
	Lateral	0.71 ± 0.18^a^	156.28 ± 10.53^a^
	Ventral	0.91 ± 0.16^a^	125 ± 9.75^ab^

Neck	Dorsal	0.85 ± 0.14^a^	134.08 ± 7.08^a^
	Lateral	0.75 ± 0.13^a^	110.43 ± 6.99^a^
	Ventral	0.57 ± 0.09^a^	117.93 ± 7.49^a^

Thorax	Dorsal	0.62 ± 0.14^a^	109.7 ± 6.34^a^
	Lateral	0.82 ± 0.18^a^	132.51 ± 13.84^ab^
	Ventral	0.89 ± 0.2^a^	165.05 ± 8.44^a^

Abdomen	Dorsal	0.98 ± 0.17^a^	157.7 ± 14.92^a^
	Lateral	1.08 ± 0.08^a^	115.5 ± 12.15^b^
	Ventral	0.38 ± 0.17^b^	160.61 ± 6.15^a^

Tail	Dorsal	0.85 ± 0.28^a^	108.04 ± 3.77^b^
	Lateral	0.74 ± 0.11^a^	113.53 ± 5.04^ab^
	Ventral	0.44 ± 0.14^a^	132.75 ± 8.95^a^

Mean value with same superscript within column does not differ significantly (p> 0.05).

**Table 2:** No. of sweat glands/mm^2^ and diameter of sweat gland in different body regions.

**Body region **	**No. of Sweat glands /mm** ^**2**^	**Sweat gland diameter (** ***μ*** **m)**
Head	0.93 ± 0.09^a^	132.30 ± 5.58^ab^
Neck	0.68 ± 0.10^a^	120.85 ± 4.28^b^
Thorax	0.76 ± .10^a^	135.80 ± 6.42^ab^
Abdomen	0.85 ± 0.10^a^	144.60 ± 7.14^a^
Tail	0.64 ± 0.12^a^	118.17 ± 3.84^b^

Mean value with same superscript within column does not differ significantly (p> 0.05).

## Data Availability

The micrometrical data was recorded during the study which has been incorporated in the article.
